# Influence of an Alkoxylation Grade of Acrylates on Shrinkage of UV-Curable Compositions

**DOI:** 10.3390/polym12112617

**Published:** 2020-11-06

**Authors:** Zbigniew Czech, Janina Kabatc, Marcin Bartkowiak, Karolina Mozelewska, Dominika Kwiatkowska

**Affiliations:** 1 International Laboratory of Adhesives and Self-Adhesive Materials, Department of Chemical Organic Technology and Polymeric Materials, Faculty of Chemical Technology and Engineering, West Pomeranian University of Technology, Pułaskiego 10, 70-322 Szczecin, Poland; marcin.bartkowiak@zut.edu.pl (M.B.); karolina_mozelewska@zut.edu.pl (K.M.); 2 Department of Organic Chemistry, Faculty of Chemical Technology and Engineering, UTP University of Science and Technology, Seminaryjna 3, 85-326 Bydgoszcz, Poland; dominika.kwiatkowska@utp.edu.pl

**Keywords:** multifunctional alkoxylated acrylates, polymerization, shrinkage, restorative materials

## Abstract

Commercially available UV curable restorative materials are composed of inorganic filler hydroxyapatite, multifunctional methacrylate, photoinitiator and alkoxylated acrylate. Especially, the application of alkoxylated monomers with different alkoxylation grade allows the reduction of polymerization shrinkage which plays the major role by application of low shrinkage composites as high quality restorative dental materials or other adhesive materials in the form of UV-polymerized self-adhesive acrylics layers (films). There are several ways to reduce polymerization shrinkage of restorative compositions, for example, by adjusting different alkoxylated acrylic monomers, which are integral part of investigated UV curable restorative composites. This article is focused on the studies of contraction-stress measured as shrinkage during UV-initiated curing of restorative composites containing various commercially available alkoxylated acrylates. Moreover, studies with experimental restorative materials and recent developments typical for UV curing technology using special photoreactive monomers are described.

## 1. Introduction

Polymerization shrinkage is still a technological problem of dental restorative composites. Polymerization shrinkage of UV curable composites is often caused by marginal and interphase defects of bonded components. The value of shrinkage phenomenon depends on the kind of applied composites: art and content of inorganic filler, and art of polymeric matrix containing multifunctional acrylates and methacrylates, radical photoinitiators and photoreactive polymeric fillers.

Dimethacrylate- or diacrylate-monomers have been applied in restorative compositions since the 1980s. Progress in filler technology, initiators systems and light sources has distinctly improved composite physical properties and extended its restorative applications [1,2]. Regardless, since early composites, the volumetric shrinkage resulting from conversion of applied dimethacrylate or methacrylate monomers into long, crosslinked polymer chains have been considered as a critical limitation that should be addressed [3,4]. The restorative composition polymerization shrinkage ranges from 2 to 6% by volume [5].

Moreover, it is well known that the modality by that light energy is used for photocuring of material influences on degree of conversion and mechanical properties of dental composites. Therefore, the properties of such materials may be “created” by using the appropriate light energy for photocuring process.

Soft light energy starts photopolymerization. The reduction of shrinkage stress [6], reduction of degree of conversion [7] may be achieved by a light modulation at the start of the light curing process (LCP). This process developed R. De Santis et al. [8] was called as the soft light energy release SLER^®^. It allows improved mechanical properties of dental restorative materials. Therefore, the photopolymerization process initiated by the soft light energy release photo-polymerization leads to reduction of about 20% the shrinkage rate and to increasing the strength of fast specimens. As a result, a more relaxed and homogeneous internal stress distribution also was observed [8].

Moreover, the chain growth and crosslinking also lead to an increase of elastic modulus [9,10]. During polymerization, there is a stage in monomer conversion referred to as an insoluble network formed within the resin phase. At this point, the elastic modulus of the composite has increased substantially, and the composite’s elastic limit has reached a level that does not allow enough plastic deformation to compensate for the reduction in volume. Beyond this stage, additional contraction may generate significant stress with the composite. If composite is bonded to cavity walls, shrinkage forces will occur, resulting in stresses on the bond between composite and restorated structure [11].

The main task of restorative adhesives is to provide a proper joint between composite fillings or composite cements and a surface of cavity walls. A good adhesive should not only withstand the mechanical forces, and in particular the shrinkage stresses of the cladding composite, but also prevent leakage along the edges of the restoration. The purpose of this article is to gather information on the properties of the chemical components that commonly make up modern adhesives [12,13].

In order to recreate the damaged material while ensuring tightness, avoid dimensional mismatches of the filling. Perfect adaptation should be achieved during set-up and also maintained during the thermal and mechanical operating cycle. From the outset, the dimensional stability of the filler material is compromised by the polymerization of the polymer matrix phase. The conversion of the monomer into a polymer network results in a closer packing of the molecules, which leads to bulk contraction [1,14]. The decrease of volume is usually marked as curing contraction or polymerization shrinkage. If the initial viscosity of material is lower, than the more monomer entities must be connected into polymer chains and networks. Although the space occupied by the filler particles does not participate in the curing shrinkage, high filler loading required monomers of low molar mass [15].

The too high shrinkage value is a main reason of use of multifunctional methacrylate monomers in UV-curable reconstructive compositions. These monomers, acting as photoreactive crosslinkers, are still main components of restorative fillings. It is well known that shrinkage is a major disadvantage of free radical photopolymerization and also influences the marginal integrity of the photo-curable system [16,17,18,19,20]. There are several ways to reduce shrinkage, for example, including monomer development (such as high mol weight dimethacrylates), reduced functionality of resins and novel polymerization methods (ring opening, step-growth, etc.).

Therefore, an application of suitable multifunctional alkoxylated monomers characterizing by low shrinkage may resolve this problem.

This paper is focused on the study of an influence of ethoxylated and propoxylated trifunctional acrylates on polymerization shrinkage of adhesive restorative materials containing hydroxylapatite and radical photoinitiator.

## 2. Materials and Methods

### 2.1. Raw Materials

Filler hydroxylapatite available from Continental Chemical (Fort Lauderdale, FL, USA) and radical photoinitiator Omnirad 127 (2-hydroxy-1-{4-[4-(2-hydroxy-2-methyl-propionyl)-benzyl]-phenyl}-2-methyl-propan-1-one) purchased from IGM Resins are presented in Table 1.

The investigated trifunctional acrylates belong to a class of ethoxylated or propoxylated monomers. Ethoxylation and propoxylation is a synthetic method of introduction of ethoxy moieties into a molecule using ethylene oxide. This reaction occurs at high temperature and pressure in presence catalyst promotion of the reaction. Starting with common base molecule trimethylolpropane triacrylate (TMPTA) and variations including three-mol propoxylated acrylate (TMP(PO)_3_TA) and three-, six- and nine-mol ethoxylated acrylates are compared (Table 2).

### 2.2. Preparing of Tested Restorative Compositions

The investigated restorative compositions, containing between 38 and 78 wt.% of hydroxylapatite, 20 to 60 wt.% of tested ethoxylated or propoxylated trifunctional acrylates and 2 wt.% of photoinitiator Ominirad 127, were coated with coat weight of 90 g/m^2^ on silicon paper and 3 min UV cured using special UV-LED curing system from Hamamatsu Photonics K.K. (Hamamatsu, Shizuoka, Japan).

### 2.3. Shrinkage Measurement

The shrinkage measurement was conducted according to specification ASTM D 2732. The resulted polymer layers were cut in size of 1 cm × 1 cm and 3 min UV cured using UV-LED curing equipment LC-L1V3 (Hamamatsu). The parameters of two UV-LED curing systems are presented in Table 3.

Dimensional changes in the restorative materials composed using EO/PO trifunctional acrylates were measured and compared after UV irradiation to the reference composition containing TMPTA. The differences in the dimensions (shrinkage S) of the irradiated samples were calculated using Equation (1):

(1) $$S[\%]=\frac{100\cdot {(A}_{0}-A)}{A_{0}}$$

where A_0_ is the initial and A is the final area of the sample after irradiation.

## 3. Results

### Shrinkage Evaluation as a Function of Alkoxylated Diacrylates Kind and Amount

**Figure 1:**
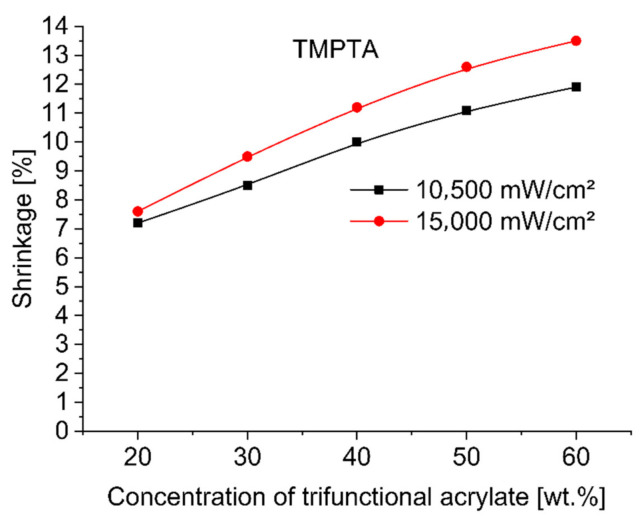
Shrinkage of UV cured restorative composition versus TMPTA concentration and UV intensity.

**Figure 2:**
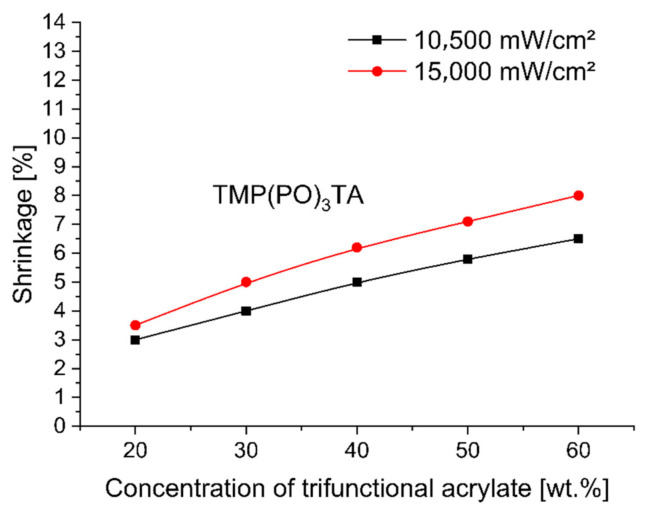
Shrinkage of UV cured restorative composition versus TMP(PO)_3_TA concentration and UV intensity.

**Figure 3:**
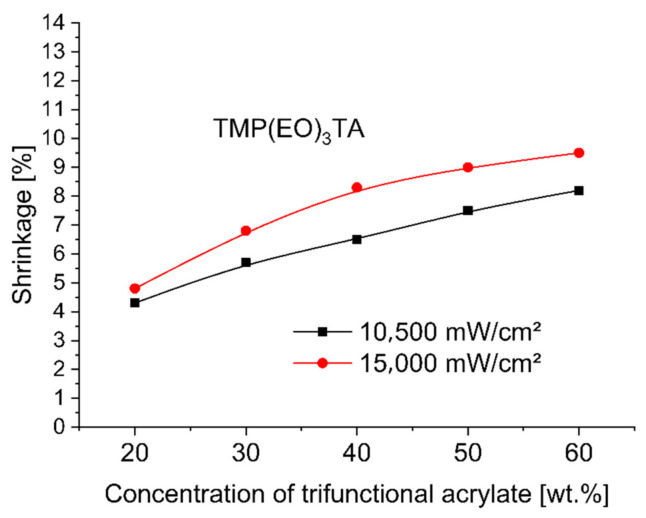
Shrinkage of UV cured restorative composition versus TMP(EO)_3_TA concentration and UV intensity.

**Figure 4:**
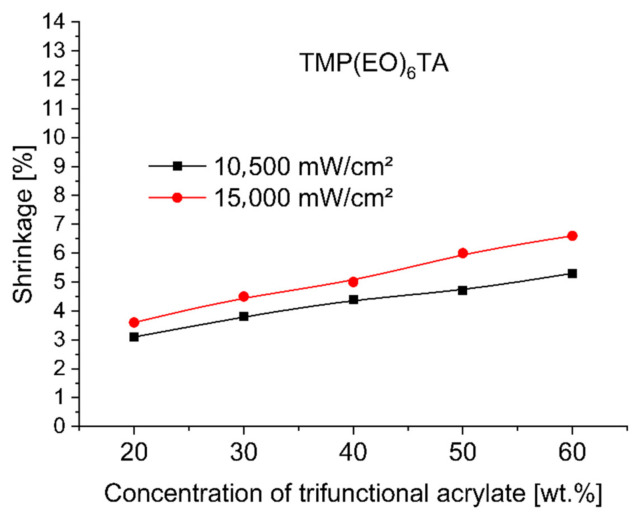
Shrinkage of UV cured restorative composition versus TMP(EO)_6_TA concentration and UV intensity.

**Figure 5:**
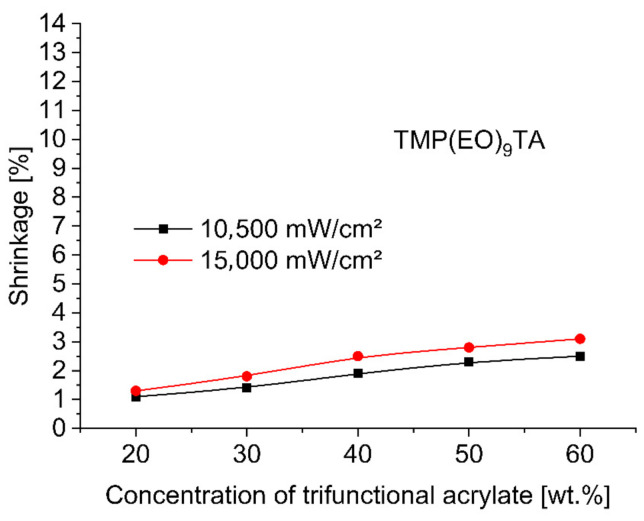
Shrinkage of UV cured restorative composition versus TMP(EO)_9_TA concentration and UV intensity.

To evaluate and compare the shrinkage run of base monomer TMPTA (trimethylolpropane triacrylate) with other trifunctional alkoxylated acrylate monomers—TMP(PO)_3_TA, TMP(EO)_3_TA, TMP(EO)_6_TA and TMP(EO)_9_TA listed in Table 2—during 3 min UV-initiated curing at 365 nm by UV power of 10,500 mW/cm^2^ and at 385 nm by UV power of 15,000 mW/cm^2^ the investigated dental compositions, containing between 38 and 78 wt.% of hydroxylapatite, 20, 30, 40, 50 and 60 wt.% of tested TMPTA, and ethoxylated or propoxylated trifunctional acrylates and 2 wt.% of photoinitiator Omnirad 127, were tested on shrinkage as a function of concentration of trifunctional acrylates studied. Figure 1, Figure 2, Figure 3, Figure 4 and Figure 5 present the shrinkage values for tested photoreactive acrylate monomers by using of UV-LED with two wavelengths (365 nm and 385 nm) and two UV intensities (10,500 mW/cm^2^ and 15,000 mW/cm^2^).

**Figure 6:**
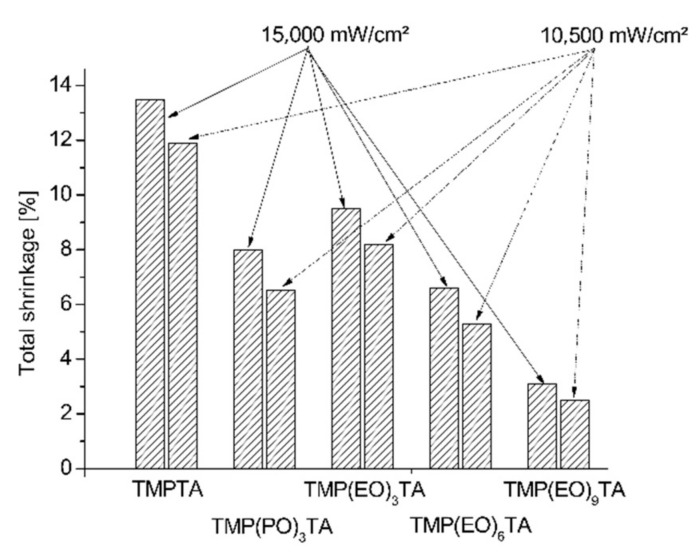
Total shrinkage of UV cured restorative materials containing investigated monomers.

The total polymerization shrinkage of restorative materials comprising investigated trifunctional acrylates shows Figure 6.

Moreover, in Table 4 are present parameters of tested restorative compositions, as well as the maximal values of shrinkage after photocuring.

**Figure 7:**
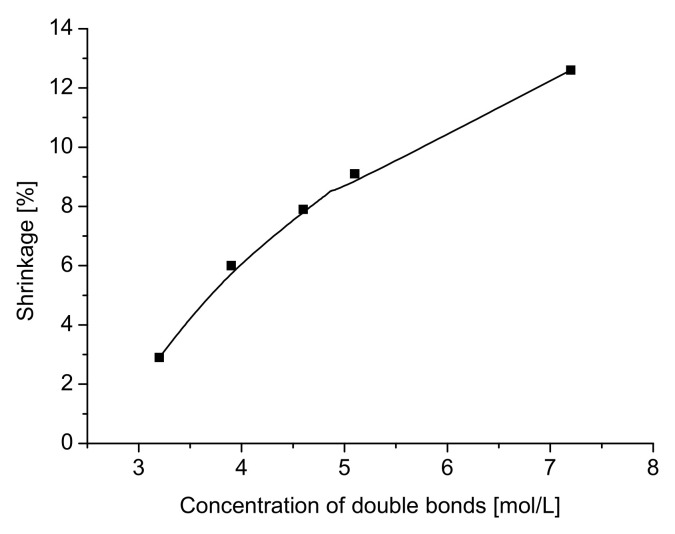
Shrinkage of dental composition versus concentration of double bonds.

The relationship between the concentration of double bonds and shrinkage is shown Figure 7 (Table 4).

## 4. Discussion

As illustrated in Figure 1, Figure 2, Figure 3, Figure 4 and Figure 5, the polymerization shrinkage of restorative matrix compositions containing trifunctional TMPTA and trifunctional propoxylated or ethoxylated acrylic monomers based on TMPTA increases with increasing concentration of mentioned trifunctional acrylates and increasing of UV radiation intensity. The changing degree of ethoxylation (TMPTA, TMP(PO)_3_TA, TMP(EO)_3_TA, TMP(EO)_6_TA and TMP(EO)_9_TA) is a convenient way to change the length of the chains of monomers as the lengths between the double bonds in the monomers. The used various diacrylates based on TMPTA with various numbers of ethoxy group with tri (TMP(EO)_3_TA), six (TMP(EO)_6_TA) and nine (TMP(EO)_9_TA) mol of ethoxy groups to prediction the effect of monomer chain lengths on polymerization shrinkage shows in Figure 6 that when the degree of ethoxylation is raised, the shrinkage obviously decreases. This effect was observed for two used UV intensities.

The concentration of double bonds (C_db_) is other important factor, that should be taken into consideration, and is defined as C_db_ (Equation (2)).

C_db_ [mol/L] = Functionality × Monomer density/Molecular weight (2)

where L is monomer chain length.

According to this equation, an increase of degree of ethoxylation leads to decrease of concentration of double bonds due to the significant increase of molecular weight. Therefore, conversion of double bonds and their concentration influence the shrinkage. Because the increase of monomer chain lengths has a more significant effect on decreasing the concentration of double bonds than on increasing conversion, shrinkage is low even at high conversion. It was also observed that the maximum shrinkage decrease was from 12.6% (ethoxylation grade 0) to 2.9% (ethoxylation grade 9), with increase the degree of ethoxylation in Table 4. Based on this, one can conclude that the monomer chain lengths effect shrinkage that is attributed to the change of the concentration of double bonds.

In addition, the reduction in the concentration of double bonds while decreasing the degree of ethoxylation causes a decrease in heat expansion. Thus, as shown in Table 4, the maximum shrinkage level moved from the expansion stage to the shrinkage stage when the degree of ethoxylation was increased. The mobility of free radicals and monomeric and pendant double bonds increases due to the formation of less strongly crosslinked networks, and the segmental diffusivity of pendant double bonds increases due to the more flexible pendant double bonds. The ethoxylation is a simple method of reducing shrinkage, because the concentration of double bonds drops significantly with increasing molecular weight of ethoxylated monomers. It was also found that the concentration of double bonds in investigated acrylate monomers influences the shrinkage of dental composition (Figure 7). It is well known that reduced functionality results in reduced shrinkage. However, it should be noted that the effect of functionality on shrinkage is very complex, because changing the functionality is always accompanied by changing both the molecular weight and the density.

## 5. Conclusions

The polymerization shrinkage of propoxylated, especially ethoxylated acrylates, depends above all on the ethoxylation grade, concentration of double bonds and UV-light intensity. Increase of chain length of monomers by higher number of ethoxy group significantly decreases the shrinkage since the obvious higher molecular weight decreases the concentration of double bonds. It is a simple way to reduce the final shrinkage. The relative concentration of double bonds also influences on both shrinkage course and shrinkage values. It was found that shrinkage decreases with the lower concentration of double bonds.

## Figures and Tables

**Table 1:** The structures of filler hydroxylapatite and photoinitiator.

Raw Material	Chemical Structure	Chemical Name
Hydroxylapatite	Ca_5_(PO_4_)_3_(OH)	mineral form of calcium apatite
Omnirad 127	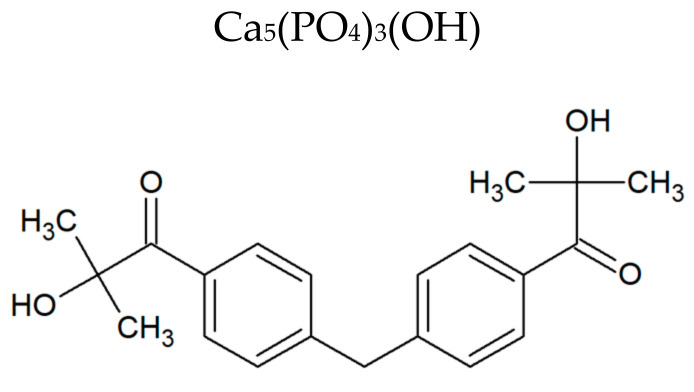	2-hydroxy-1-{4-[4-(2-hydroxy-2-methyl-propionyl)-benzyl]-phenyl}-2-methyl-propan-1-one

**Table 2:** Trifunctional alkoxylated acrylates investigated in this study.

Monomer	Chemical Structure	Producer	M_W_ [Dalton]
Miramer M300	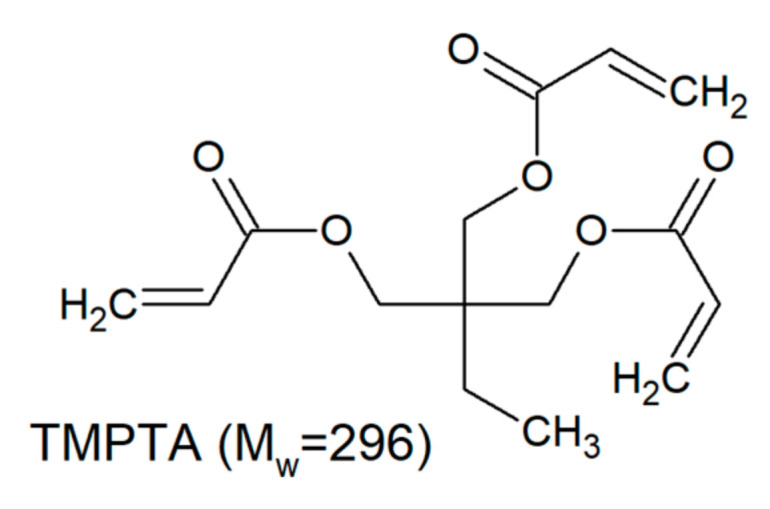	Rahn USA (Aurora, IL)	296
Miramer M360	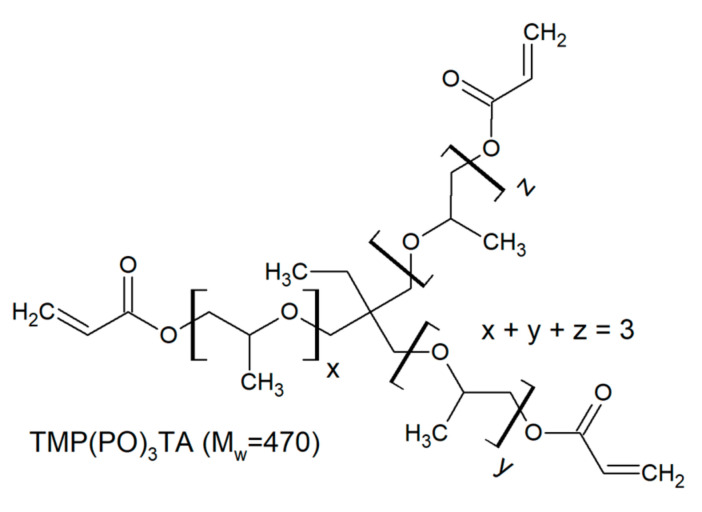	Rahn USA (Aurora, IL)	470
Miramer M3130	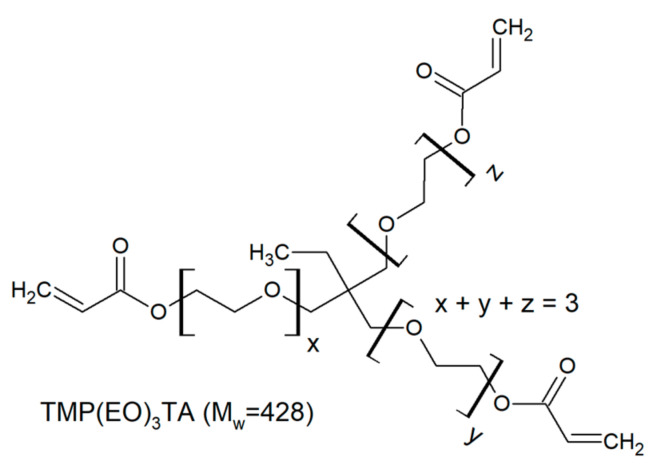	Rahn USA (Aurora, IL)	428
Miramer M3190	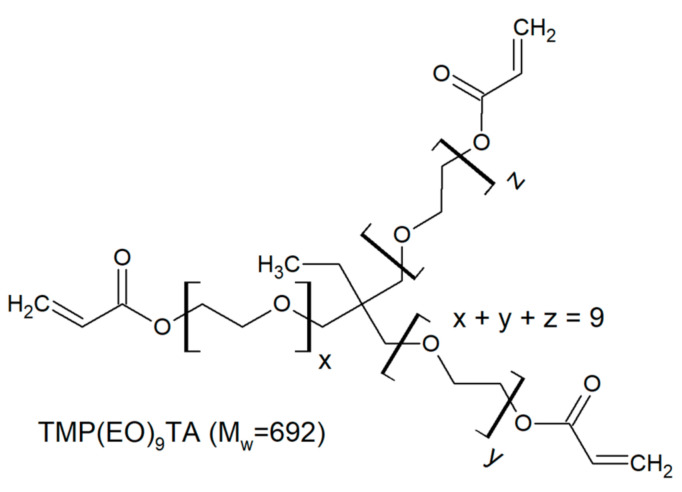	Rahn USA (Aurora, IL)	692

**Table 3:** Important parameters of UV-LED curing systems applied in this work.

Parameter	High Power Type 365 nm	High Power Type 385 nm
UV irradiation intensity	10,500 mW/cm^2^	15,000 mW/cm^2^
Peak wavelength	365 ± 5	385 ± 5

**Table 4:** Monomers parameters and shrinkage of tested restorative compositions.

Monomer	Functionality	Molecular Weight [kg/kmol]	Density at 25 °C [kg/m^3^]	Concentration of Double Bonds C_db_ [mol/L]	Maximal Shrinkage after UV Curing [%]
TMPTA	3	296	1060	7.2	12.6
TMP(PO)_3_TA	3	470	1070	4.6	7.9
TMP(EO)_3_TA	3	428	1090	5.1	9.1
TMP(PO)_6_TA	3	560	1100	3.9	6.0
TMP(PO)_9_TA	3	692	1110	3.2	2.9
